# Development of *ptxD*/Phi as a new dominant selection system for genetic manipulation in *Cryptococcus neoformans*

**DOI:** 10.1128/spectrum.01618-24

**Published:** 2024-11-20

**Authors:** Muthita Khongthongdam, Tanaporn Phetruen, Sittinan Chanarat

**Affiliations:** 1 Laboratory of Molecular Cell Biology, Department of Biochemistry and Center for Excellence in Protein and Enzyme Technology, Faculty of Science, Mahidol University, Bangkok, Thailand; 2 Laboratory of Medical Molecular Mycology, Department of Biochemistry, Faculty of Science, Mahidol University, Bangkok, Thailand; Universita degli Studi di Modena e Reggio Emilia, Modena, Italy

**Keywords:** *Cryptococcus neoformans*, genetic manipulation, gene deletion, selectable marker, *ptxD *gene, phosphite dehydrogenase, CRISPR-Cas9, virulence factors

## Abstract

*Cryptococcus neoformans* is a globally distributed pathogenic fungus posing a significant threat to immunocompromised individuals, particularly those with HIV/AIDS. Effective genetic manipulation tools are essential for understanding its biology and developing new therapies. However, current genetic tools, including the variation of versatile selectable markers, are limited. This study develops and validates the phosphite dehydrogenase gene (*ptxD*)/phosphite (Phi) selection system as a non-antibiotic selectable marker for genetic manipulation in *C. neoformans*. A codon-optimized *ptxD* gene from *Pseudomonas stutzeri* was cloned under the *TEF* promoter. Using the transient CRISPR-Cas9 coupled with electroporation system, we integrated the *ptxD* gene into the *C. neoformans* genome and assessed the impact of *ptxD* integration on cell growth and virulence factors. The *ptxD*/Phi system effectively selected transformed cells on Phi-containing media. Growth assays showed that *ptxD* integration did not adversely affect cell growth or key virulence factors, including pleomorphism, capsule size, and melanin production. Additionally, we successfully disrupted the *ADE2* gene using this system, confirming its applicability for gene deletion. Taken together, the *ptxD*/Phi system provides a robust and versatile tool for genetic manipulation in *C. neoformans*, facilitating further research into its biology and pathogenicity.

**IMPORTANCE:**

*Cryptococcus neoformans* is a type of fungus that can cause serious illnesses in people who have weakened immune systems, like those with HIV/AIDS. To better study this fungus and find new treatments, scientists need tools to change its genes in precise ways. However, the current tools available for this are somewhat limited. This research introduces a new tool called the phosphite dehydrogenase gene/phosphite system, which does not rely on antibiotics to work. It uses a gene from a different bacterium that helps select and grow only the fungus cells that have successfully incorporated new genetic information. This is particularly useful because it does not interfere with the normal growth of the fungus or the features that make it harmful (like its ability to change shape or produce protective coatings). By making it easier and more effective to manipulate the genetics of *C. neoformans*, this tool opens up new possibilities for understanding how this fungus operates and for developing therapies to combat its infections. This is crucial for improving the treatment of infections in vulnerable populations.

## INTRODUCTION

*Cryptococcus neoformans* poses a significant threat to human health globally, particularly affecting immunocompromised individuals. This opportunistic fungus can cause cryptococcosis, with infections primarily occurring in the lungs but often leading to severe fungal meningitis and encephalitis, especially in AIDS patients (1, 2). *C. neoformans* is known for its ability to spread to the central nervous system, causing meningoencephalitis, a potentially fatal condition (3, 4). While infections with this fungus are rare in individuals with healthy immune systems, its facultative intracellular nature allows it to survive within host cells, leading to latency, disseminated disease, and resistance to antifungal treatments (5, 6). The traversal of the blood-brain barrier by *C. neoformans* is a key factor in the pathogenesis of meningitis, although the precise mechanisms of this process are still not fully understood. The ability of the fungus to cause serious complications such as systemic infections, fatal meningitis, and a range of neurological issues underscores its global impact on human health.

*C. neoformans* was ranked as the top critical priority fungal pathogen on the World Health Organization (WHO) Fungal Priority Pathogens List (7, 8). This ranking was based on several factors, including its high average case fatality rate, particularly in immunocompromised individuals such as those with HIV/AIDS. *C. neoformans* is estimated to cause over 200,000 deaths annually, primarily from cryptococcal meningitis (9). The fungus is globally distributed and has shown an increasing incidence in the past decade. Complications from *C. neoformans* infections can be severe, including disseminated disease and central nervous system involvement leading to meningoencephalitis. Additionally, there are growing concerns about the emergence of antifungal resistance in *C. neoformans* isolates, which can complicate treatment (10, 11). The WHO’s critical priority ranking for this pathogen underscores the urgent need for improved diagnostics, therapeutics, and public health interventions to combat the significant global burden of cryptococcosis.

Despite its significant impact on human health, research on *C. neoformans* is hindered by the lack of diverse genetic manipulation tools. Currently, the main selectable markers used for genetic manipulation in *Cryptococcus* species include the *URA5* gene, which encodes orotate phosphoribosyltransferase and allows for selection on media lacking uracil, and the *ADE2* gene, which encodes phosphoribosylaminoimidazole carboxylase and allows for selection on media lacking adenine (12–16). Additionally, the *amdS* gene, which encodes acetamidase, is used for selection on media containing acetamide as the sole nitrogen source (17). Dominant selectable markers such as the nourseothricin resistance gene (*NAT*) and the hygromycin B resistance gene (*HPH*) confer resistance to their respective antibiotics and are also commonly used (18, 19). However, these selectable markers have limitations. For instance, the use of auxotrophic markers requires the availability of auxotrophic strains, and dominant markers like antibiotic resistance genes are not always preferable due to concerns over antibiotic use. Additionally, some markers can alter the phenotypes and virulence of the fungus, making them unsuitable for studying its physiological virulence (20).

To enhance genetic manipulation in *C. neoformans*, there is a need for the development of new selectable markers and molecular techniques. In this current work, we introduce a new gene marker, *ptxD*, from *Pseudomonas stutzeri*, for genetic manipulation in *C. neoformans*. This marker is selectable on media lacking phosphate but containing phosphite. When integrated into the fungus, the *ptxD* marker does not alter its overall phenotypes, including cell growth and virulence factors such as capsule size, cell pleomorphism, and melanin production. Taken together, this paper describes a novel non-antibiotic selectable marker for use in the genetic manipulation of *C. neoformans*, with the aim of enhancing research and development in this pathogenic fungus.

## MATERIALS AND METHODS

### Yeast strains and culture

Yeast strains, plasmids, and primers are listed in Tables S1 to S3, respectively. *C. neoformans* KN99α strain was stored as glycerol stocks at −80°C. Upon recovery, the cultures were incubated for up to 3 days at 30°C on YPD (1% yeast extract [Himedia; RM027], 2% peptone [Himedia; RM001], and 2% glucose [Sigma-Aldrich]). For phosphate limitation studies, synthetic complete (SC) medium (either 6.7 g/L yeast nitrogen without amino acids and without phosphate supplemented with KCI [Formedium; CYN6701] or yeast nitrogen without amino acids [Formedium; CYN0401], 2% glucose [Sigma-Aldrich], 1.394 g/L Kaiser SC mixture drop-out: -HIS-LEU-TRP-URA [Formedium; DSCK1027], 2 g/L histidine [Himedia; GRM050], 2 g/L tryptophan [Himedia; GRM067], 2 g/L uracil [Glentham Life Sciences; GK0306], and 3 g/L leucine [Phyto Technology Laboratories; L574]) was modified to create a phosphorus-free defined minimal medium (MM) by omitting phosphate from the standard components of the SC medium. For dependency testing, either 7.35 mM phosphate (Pi) or phosphite (Phi) (Sigma-Aldrich; 04283) was supplemented as required.

### Cloning of pFA6a-Cno-*ptxD* plasmid

The *ptxD* gene, codon-optimized for *C. neoformans* (Cno) and originally derived from *Pseudomonas stutzeri*, was chemically synthesized by GenScript. This synthesized gene fragment was then cloned into the pFA6a plasmid using *in vivo* assembly techniques (21).

### Transformation of *Cryptococcus neoformans* by electroporation using a transient CRISPR-Cas9 expression

Transformation of *C. neoformans* was performed according to the TRACE (transient CRISPR-Cas9 coupled with electroporation) protocol with slight modifications (22). To achieve specific integration of the *ptxD* gene into the SH2 (safe haven 2) or *ADE2* loci, *C. neoformans* strain KN99α was subjected to electroporation with 700 ng of the Cas9 cassette, 2 µg of the *ptxD* gene cassette, and 700 ng of either the SH2 or *ADE2* single guide RNA (sgRNA) cassette. Specific primer pairs were used to amplify the sgRNA scaffold, which incorporates the 6T terminator and the U6 promoter, from plasmids pCnCas9:U6-gRNA and pBHM2329, respectively. The sgRNA-expressing cassettes underwent two rounds of PCR amplification, featuring guide sequences that target SH2 or *ADE2* for homologous recombination and precise Cas9 guidance to these loci. Additionally, specific primers amplified the *CAS9* gene from the pBHM2403 plasmid. The *ptxD* gene cassette from the pFA6a-Cno-*ptxD* plasmid was also amplified, ensuring the preparation of all components for successful genomic integration.

For transformation, 5 mL of YPD liquid medium was inoculated and shaken at 250 rpm overnight. The overnight culture was diluted to an optical density at 600 nm (OD_600_) of 0.2 and transferred to a 250-mL flask containing 50 mL of YPD. This culture was then incubated at 30°C with shaking at 250 rpm for at least 4 hours until it reached the log phase (OD_600_ = 0.6–1). To halt cell growth, the flask was placed on ice for 15 minutes. The cultures were then centrifuged at 4,000 rpm for 5 minutes at 4°C in a 50-mL Falcon tube, and the YPD medium was discarded. The cell pellets were resuspended in 8 mL of sterile water, and 1 mL of 10× TE buffer (10 mM Tris, pH 8, and 1 mM EDTA) with 1 M lithium acetate was added and mixed thoroughly. This mixture was incubated at 30°C with shaking at 180 rpm for 45 minutes. Then, 0.25 mL of 1 M dithiothreitol (DTT) was added, and the mixture was incubated again for 15 minutes at the same temperature and shaking speed, followed by the removal of the supernatant. The cell pellet was then dissolved in 5 mL of sterile, ice-cold sorbitol and centrifuged again at 4,000 rpm for 5 minutes at 4°C to remove the supernatant. Finally, the cell pellet was resuspended in 50 µL of sterile, ice-cold sorbitol (23).

For the transformation, a 1.5-mL microcentrifuge tube was prepared by combining 5 µL of the DNA fragment mixture with 45 µL of competent cells to ensure thorough mixing. This DNA-cell mixture was then transferred to an electroporation cuvette. The mixture included approximately 700 ng of the Cas9, 700 ng of the sgRNA (targeting SH2 or *ADE2*), and 2 µg of the *ptxD* cassettes. The BioRad Gene Pulser was set to 0.45 kV, 450 Ω resistance, and 125 µF capacitance. After electroporation, the cells were allowed to recover in YPD at 30°C for 2 hours. Finally, the transformed cells were plated on SC-Pi+Phi agar plates to facilitate selection (22, 24).

### Evaluation of virulence factors

#### Growth curves

*C. neoformans* strain KN99α was cultured overnight in YPD medium. Prior to the experiment, the cells were harvested by centrifugation and washed once with MilliQ water. The cells were then diluted to an OD_600_ of 0.2 in various media: SC, SC-Pi (synthetic complete without phosphate), and SC-Pi+Phi (synthetic complete without phosphate and supplemented with phosphite). These cell suspensions were distributed into 96-well plates. The plates were incubated at 30°C with shaking, and cell viability was monitored. OD_600_ was measured every hour for a total of 24 hours using a microplate reader to assess growth curves.

#### Capsule induction

Cells were cultured overnight in YPD medium. Prior to the experiments, cells were harvested by centrifugation and washed once with MilliQ water. The washed cells were diluted to an OD_600_ of 0.2 and inoculated into 3 mL of the following media: YPD, 1/10 Sabouraud dextrose broth (SDB) supplemented with 50 mM 3-(N-morpholino)propanesulfonic acid (MOPS) at pH 7.3, and Dulbecco’s modified Eagle medium (DMEM). These samples were placed in six-well plates and incubated at 37°C in a CO_2_ environment for 3 and 5 days. For measuring capsule thickness and cell size, 5 µL of the cell suspension from each well was mixed with India ink and examined under a light microscope at 100× magnification. Random fields were selected for observation and analysis using the ImageJ software (25).

#### Melanin pigmentation

Cells were initially cultured overnight in YPD medium. Subsequently, the cells were diluted to an OD_600_ of 0.2 in MM. These cells were then spotted onto MM supplemented with 1 mM l-3,4-dihydroxyphenylalanine (L-DOPA) and incubated at 30°C for 1–3 days to assess melanin production. For comparative analysis, another set of cells was spotted onto MM without L-DOPA supplementation. This setup allowed for the examination of melanin pigmentation under varying conditions, facilitating a detailed analysis of the effects of L-DOPA on melanin synthesis in *C. neoformans* (26, 27).

## RESULTS AND DISCUSSION

### Evaluation of phosphate dependency in *C. neoformans*

**Fig 1:**
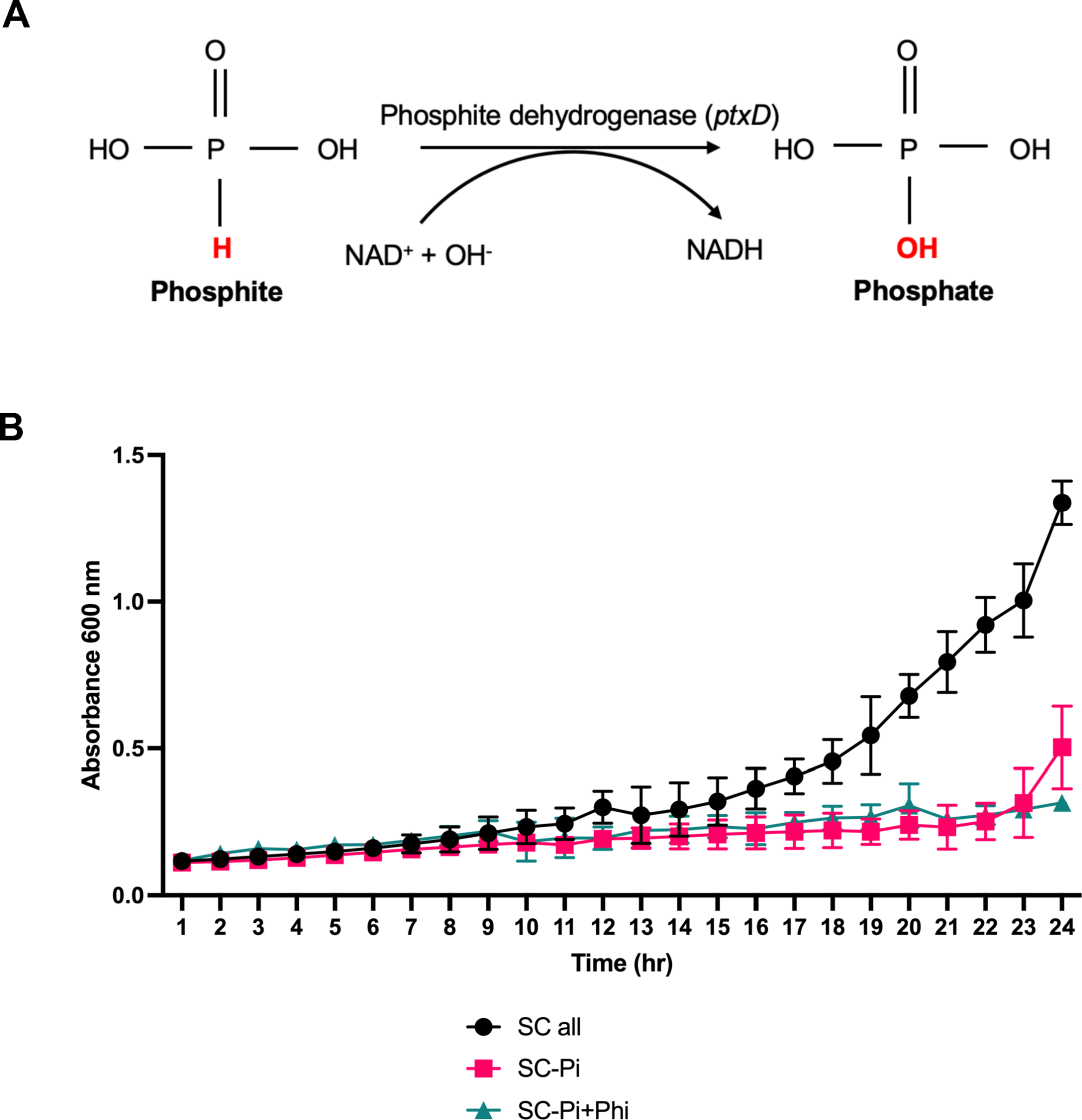
Impact of phosphate on *C. neoformans* growth. (**A**) Schematic of the biochemical reaction catalyzed by the *ptxD* gene product, which converts phosphite into bioavailable phosphate. (**B**) *C. neoformans* cultures in SC complete media, SC-Pi (phosphate-free) media, and SC-Pi+Phi (phosphate-free supplemented with phosphite) media over 24 hours. Growth rates in SC media lacking phosphate are noticeably slower than the condition with phosphate.

To identify a new candidate for a selectable marker in *C. neoformans*, the *ptxD* gene was considered due to its numerous advantages. The *ptxD* gene, originating from bacteria, encodes the enzyme phosphite dehydrogenase, which catalyzes the oxidation of phosphite to phosphate, coupled with the reduction of NADP+ to NADPH (Fig. 1A) (28). This biochemical reaction allows transformed cells to grow on a medium containing Phi as the sole phosphorus source. The *ptxD*/Phi selection system offers several benefits over traditional selectable markers. It is a non-antibiotic-based system, reducing concerns about potential environmental risks and gene flow. Additionally, Phi is a non-metabolizable phosphorus source, so only transformed cells expressing *ptxD* can utilize it for growth, enabling efficient selection. This system is cost-effective and straightforward, as it does not require the addition of selective agents that can negatively impact cell proliferation and differentiation. Therefore, we propose using the *ptxD* gene, combined with Phi as the sole phosphorus source, as an effective and efficient selectable marker system for genetic transformation, with potential applications in *C. neoformans*.

Before exploring whether the *ptxD* gene can be used as a selectable marker in *C. neoformans*, it is essential to determine if the fungus can survive without phosphate in minimal media. This step is crucial because the effectiveness of the *ptxD*/Phi selection system relies on the inability of untransformed cells to grow in the absence of phosphate. By confirming that *C. neoformans* requires phosphate for growth and cannot utilize phosphite, we can ensure that only cells transformed with the *ptxD* gene will grow when phosphate is not available. This validation is necessary to enable efficient selection of genetically modified cells. To this end, we cultured the *C. neoformans* KN99ɑ wild-type strain in synthetic complete media under different conditions: lacking phosphate (SC-Pi), lacking phosphate but containing phosphite (SC-Pi+Phi), and containing all nutrients (SC+all) (Fig. 1B). As expected, cells did not grow well in the phosphate-absent media, supporting the potential use of the *ptxD*/Phi system as a viable selectable marker.

### Evaluating the impact of *ptxD* integration on cell growth of *C. neoformans*

**Fig 2:**
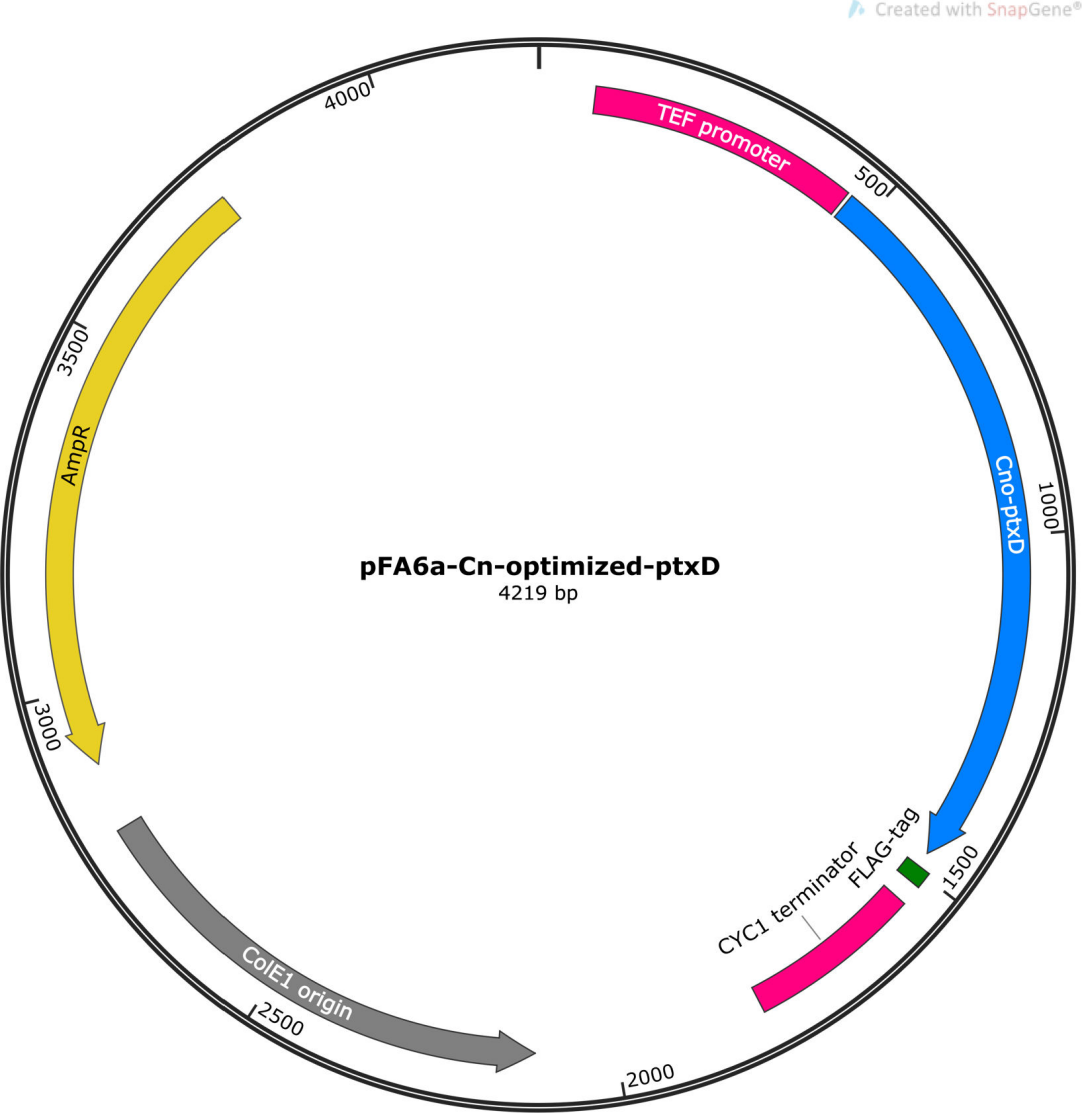
Schematic of the pFA6a-Cno-*ptxD* plasmid. This diagram illustrates the pFA6a-Cno-*ptxD* plasmid used in transformation experiments, constructed via *in vivo* assembly. The plasmid includes the *ptxD* gene, which was synthesized and codon-optimized for optimal expression in *Cryptococcus neoformans*. The plasmid map was generated using SnapGene software (Dotmatics; available at snapgene.com).

**Fig 3:**
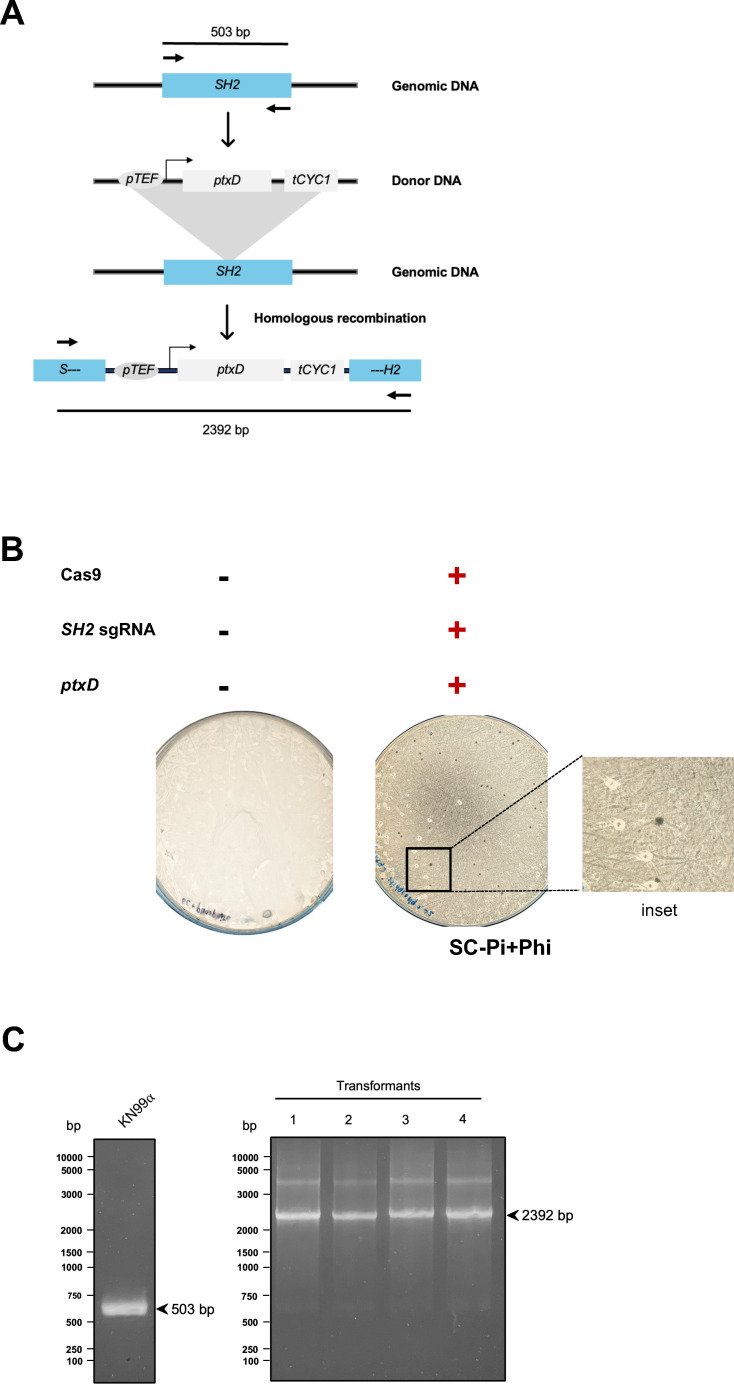
CRISPR-Cas9 mediated transformation experiment. (**A**) Schematic representation of integration strategy using CRISPR-Cas9 mediated transformation. (**B**) Transformation plates for *ptxD* insertion at the SH2 locus on SC-Pi+Phi media, highlighting successful *ptxD* gene integration. (**C**) Colony PCR analysis for *ptxD* gene integration shows individual colonies tested for *ptxD* integration at the SH2 locus. Comparison with control strain KN99α reveals distinct band sizes, indicating successful integration.

After confirming the phosphate dependency in *C. neoformans*, we constructed a codon-optimized version of the *ptxD* gene to ensure optimal expression in the fungal system. This optimized gene was cloned under the control of the *TEF* promoter into a plasmid, which serves as a PCR template for subsequent transformations (Fig. 2). Next, to integrate the pTEF-*ptxD* gene into the SH2 locus of the *C. neoformans* genome, we employed the TRACE system (Fig. 3A). Following integration, cells were plated on SC-Pi+Phi agar to assess their viability and growth. This experimental strategy aimed to verify the effectiveness of the *ptxD* system as a selectable marker in the pathogenic yeast. As a result, we successfully obtained transformants in a construct-dependent and Phi-dependent manner (Fig. 3B; Table S4). Moreover, colony PCR was able to validate the correct integration into the specific locus (Fig. 3C; Fig. S1A and B). These findings suggest that the *ptxD*/Phi marker system is effective for selection and can be accurately inserted at the intended genomic locus.

**Fig 4:**
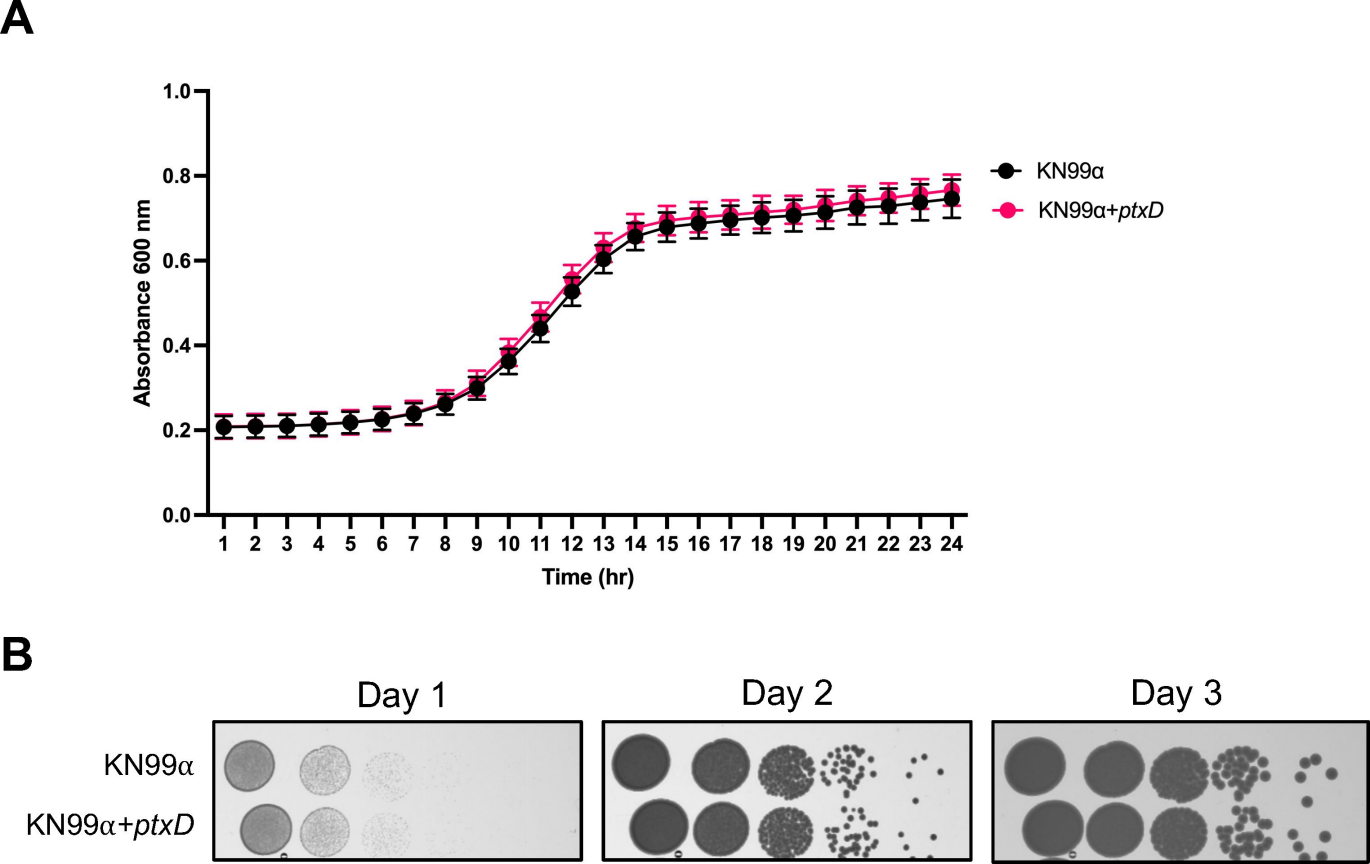
Integration of *ptxD* gene does not affect growth of *C. neoformans*. (**A**) Growth curves for the *ptxD*-integrated strain and the control strain KN99α in YPD medium, with OD_600_ measurements taken hourly over a 24-hour period. (**B**) Colonies of both the *ptxD*-integrated strain and control strain KN99α were spotted on YPD agar plates and incubated at 30°C for 3 days. Observations indicate no significant difference in growth rates between the *ptxD*-integrated strain and the control strain in YPD medium.

To validate the *ptxD*/Phi system as an effective selectable marker, we evaluated whether cells harboring the *ptxD* gene maintained their normal growth and virulence factors. Firstly, we compared the growth of the original KN99ɑ strain, which had not been genetically modified, with two independent colonies of KN99ɑ that harbored the *ptxD* gene integrated at the SH2 locus—a genomic site previously demonstrated not to affect the phenotypes of the fungus (29, 30). Growth curve analysis showed that cells containing the *ptxD* gene grew comparably to the original wild-type cells, indicating that the gene does not unfavorably affect the growth of *C. neoformans* in both solid and liquid media, across all temperature tested (16°C, 25°C, 30°C, and 37°C) (Fig. 4A and B; Fig. S2A and B, S3A and B). Note that the growth of cells in SC-Pi+Phi was comparable to that in YPD, further supporting that the *ptxD* gene does not negatively affect fungal growth (Fig. S4).

### Evaluating the impact of *ptxD* integration on virulence factors of *C. neoformans*

**Fig 5:**
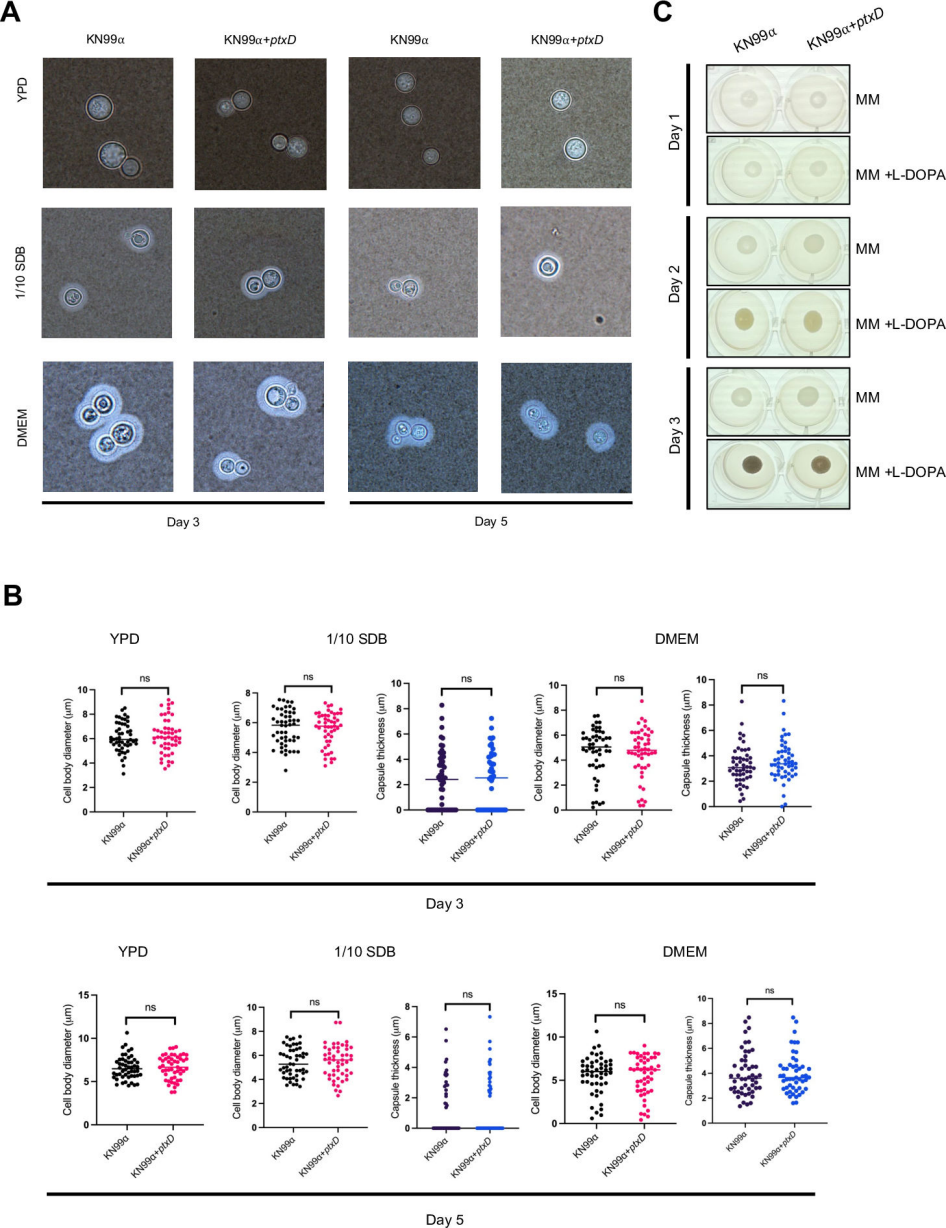
Integration of *ptxD* gene does not affect virulence factors of *C. neoformans*. (**A**) Capsule induction in various media. The *ptxD*-integrated strain and the control strain KN99α were subjected to capsule induction in different media: YPD (control medium), 1/10 SDB (for capsule induction), and DMEM (for capsule and cell differentiation). Capsule formation was evaluated after incubation at 37°C with CO_2_ for 3 and 5 days. (**B**) Quantification of (A): measurements and analyses of capsule and cell sizes were conducted, showing no significant differences between the *ptxD*-integrated strain and the control strain KN99α across YPD, 1/10 SDB, and DMEM media. (**C**) The melanin production capability of *C. neoformans* was assessed in MM supplemented with L-DOPA, with MM without L-DOPA serving as a control. The integration of the *ptxD* gene did not influence melanin production in *C. neoformans*, maintaining similar levels to the wild-type control strain KN99α in both supplemented and non-supplemented MM.

For a selectable marker to be suitable for use in pathogenic organisms, it is crucial that the marker gene does not influence their virulence. In *C. neoformans*, several key virulence factors need to be considered, and the *ptxD*/Phi system must meet this criterion. We evaluated the impact of *ptxD* integration on essential virulence factors such as pleomorphism, capsule size, and melanin production. These factors were compared between the original KN99ɑ strain and strains harboring the *ptxD* gene (Fig. 5A through C). We found that none of the virulence factors were altered by the integration of the *ptxD* gene, indicating that this *ptxD*/Phi selection system could effectively serve as a selectable marker in *C. neoformans* without compromising its pathogenic properties.

### Utilizing the *ptxD*/Phi selection system for gene deletion in *C. neoformans*: disruption of the *ADE2* gene

**Fig 6:**
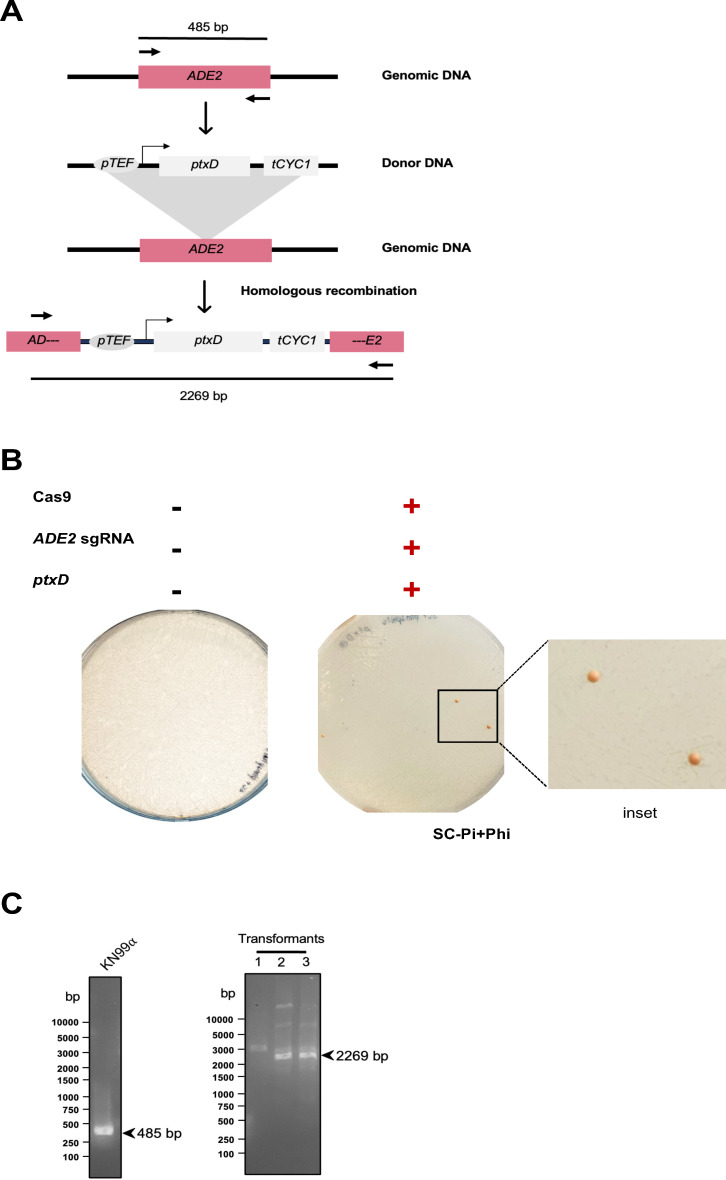
The *ptxD* integration can be used to disrupt target genes in *C. neoformans*. (**A**) Schematic representation of integration strategy using CRISPR-Cas9 mediated *ADE2* gene disruption. (**B**) Transformation plates showing the insertion of the *ptxD* gene into the *ADE2* locus. The integration of *ptxD* is displayed on SC-Pi+Phi media plates, showing pink colonies of *ADE2*-disrupted cells. (**C**) Colony PCR analysis confirming the successful integration of the *ptxD* gene into the *ADE2* locus. The control strain KN99α exhibits a different band size, distinguishing wild-type colonies from those successfully modified with the *ptxD* gene insertion.

To explore further potential applications of the selection system, we employed it for disrupting the *ADE2* gene, encoding an enzyme involved in purine biosynthesis. Disruption of the *ADE2* gene in yeast leads to a distinct phenotype where cells accumulate a pigment in the vacuole, resulting in the formation of red colonies instead of the typical white colonies. Utilizing the TRACE method, we designed a sgRNA targeting the coding sequence of the *ADE2* gene. The *ptxD* cassette, along with the sgRNA and *CAS9* gene cassettes, was co-electroporated into *C. neoformans* KN99ɑ cells. After 4–5 days of incubation on Phi agar plates, several colonies were obtained. Transformants were subjected to colony PCR to confirm the disruption of the *ADE2* gene (Fig. 6A). Subsequently, phenotype analysis revealed that all colony-PCR positive cells exhibited a pink/red coloration (Fig. 6B and C; Fig. S1A). These results demonstrate the successful use of the *ptxD*/Phi selection system for genetic manipulation in the fungus, highlighting its versatility and efficacy in facilitating gene manipulation processes in *C. neoformans*.

### Conclusions

In this study, we developed and validated the *ptxD*/Phi system as a novel selectable marker for genetic manipulation in *Cryptococcus neoformans*. Our findings demonstrate that the codon-optimized *ptxD* gene, when integrated into the fungal genome under the control of the *TEF* promoter, allows for the effective selection on phosphite-containing media. This system offers several advantages, including being non-antibiotic based and cost-effective, and does not require selective agents that could negatively impact cell proliferation and differentiation. We confirmed the phosphate dependency of *C. neoformans* and successfully integrated the *ptxD* gene into the SH2 locus using the TRACE system. The transformants showed no alteration in growth or key virulence factors such as pleomorphism, capsule size, and melanin production. Additionally, we demonstrated the applicability of the *ptxD*/Phi system for gene deletion by disrupting the *ADE2* gene, resulting in a distinct phenotype that confirmed successful genetic manipulation.

Although it is theoretically possible that the *ptxD* gene product might have off-target effects that could alter the yeast’s phenotype, no off-target effects of this enzyme have been identified to date. Furthermore, natural phosphite levels are significantly lower than phosphate, and the conversion of phosphite to phosphate is known to be insufficient to impact biological processes (31, 32). Therefore, we conjecture that using *ptxD* as a selectable marker will not affect the yeast’s phenotype, supporting its use in further research and development aimed at understanding and combating this significant fungal pathogen.

Overall, the *ptxD*/Phi system provides a robust and versatile tool for genetic studies in *C. neoformans*, facilitating further research and development.

## Data Availability

The complete plasmid sequence of pFA6a-Cno-*ptxD* has been deposited in the NCBI database under the accession number PP987167.1.

## SUPPLEMENTAL MATERIAL

Supplemental material spectrum.01618-24-s0001.pdf Tables S1 to S4; Fig. S1 to S4.

ASM does not own the copyrights to Supplemental Material that may be linked to, or accessed through, an article. The authors have granted ASM a non-exclusive, world-wide license to publish the Supplemental Material files. Please contact the corresponding author directly for reuse.

## References

[1] Skolnik K, Huston S, Mody CH. 2017. Cryptococcal lung infections. Clin Chest Med 38:451–464. doi:10.1016/j.ccm.2017.04.007 28797488

[2] Rodrigues ML, Alviano CS, Travassos LR. 1999. Pathogenicity of Cryptococcus neoformans: virulence factors and immunological mechanisms. Microbes Infect 1:293–301. doi:10.1016/s1286-4579(99)80025-2 10602663

[3] Hamed MF, Enriquez V, Munzen ME, Charles-Niño CL, Mihu MR, Khoshbouei H, Alviña K, Martinez LR. 2023. Clinical and pathological characterization of central nervous system cryptococcosis in an experimental mouse model of stereotaxic intracerebral infection. PLoS Negl Trop Dis 17:e0011068. doi:10.1371/journal.pntd.0011068 36656900 PMC9888703

[4] González-Duarte A, Higera Calleja J, Mitre VG, Ramos GG. 2009. Simultaneous central nervous system complications of C. neoformans infection. Neurol Int 1:e22. doi:10.4081/ni.2009.e22 21577360 PMC3093228

[5] Alanio A. 2020. Dormancy in Cryptococcus neoformans: 60 years of accumulating evidence. J Clin Invest 130:3353–3360. doi:10.1172/JCI136223 32484459 PMC7324190

[6] Coelho C, Bocca AL, Casadevall A. 2014. The intracellular life of Cryptococcus neoformans*.* Annu Rev Pathol 9:219–238. doi:10.1146/annurev-pathol-012513-104653 24050625 PMC5127716

[7] Casalini G, Giacomelli A, Antinori S. 2024. The WHO fungal priority pathogens list: a crucial reappraisal to review the prioritisation. Lancet Microbe 5:717–724. doi:10.1016/S2666-5247(24)00042-9 38608682

[8] World Health Organization. 2022. WHO fungal priority pathogens list to guide research, development and public health action. World Health Organization.

[9] Rajasingham R, Smith RM, Park BJ, Jarvis JN, Govender NP, Chiller TM, Denning DW, Loyse A, Boulware DR. 2017. Global burden of disease of HIV-associated cryptococcal meningitis: an updated analysis. Lancet Infect Dis 17:873–881. doi:10.1016/S1473-3099(17)30243-8 28483415 PMC5818156

[10] Bermas A, Geddes-McAlister J. 2020. Combatting the evolution of antifungal resistance in Cryptococcus neoformans. Mol Microbiol 114:721–734. doi:10.1111/mmi.14565 32697029

[11] Melhem MSC, Leite Júnior DP, Takahashi JPF, Macioni MB, Oliveira L de, de Araújo LS, Fava WS, Bonfietti LX, Paniago AMM, Venturini J, Espinel-Ingroff A. 2024. Antifungal resistance in cryptococcal infections. Pathogens 13:128. doi:10.3390/pathogens13020128 38392866 PMC10891860

[12] Perfect JR, Toffaletti DL, Rude TH. 1993. The gene encoding phosphoribosylaminoimidazole carboxylase (ADE2) is essential for growth of Cryptococcus neoformans in cerebrospinal fluid. Infect Immun 61:4446–4451. doi:10.1128/iai.61.10.4446-4451.1993 8406836 PMC281178

[13] Edman JC, Kwon-Chung KJ. 1990. Isolation of the URA5 gene from Cryptococcus neoformans var. neoformans and its use as a selective marker for transformation. Mol Cell Biol 10:4538–4544. doi:10.1128/mcb.10.9.4538-4544.1990 2201894 PMC361041

[14] Wang P, Cardenas ME, Cox GM, Perfect JR, Heitman J. 2001. Two cyclophilin A homologs with shared and distinct functions important for growth and virulence of Cryptococcus neoformans. EMBO Rep 2:511–518. doi:10.1093/embo-reports/kve109 11415984 PMC1083903

[15] Lin X, Chacko N, Wang L, Pavuluri Y. 2015. Generation of stable mutants and targeted gene deletion strains in Cryptococcus neoformans through electroporation. Med Mycol Open Access 53:225–234. doi:10.1093/mmy/myu083 PMC4574871 25541555

[16] Huang MY, Joshi MB, Boucher MJ, Lee S, Loza LC, Gaylord EA, Doering TL, Madhani HD. 2022. Short homology-directed repair using optimized Cas9 in the pathogen Cryptococcus neoformans enables rapid gene deletion and tagging. Genetics 220:iyab180. doi:10.1093/genetics/iyab180 34791226 PMC8733451

[17] Erpf PE, Stephenson CJ, Fraser JA. 2019. amdS as a dominant recyclable marker in Cryptococcus neoformans. Fungal Genet Biol 131:103241. doi:10.1016/j.fgb.2019.103241 31220607

[18] Cox GM, Toffaletti DL, Perfect JR. 1996. Dominant selection system for use in Cryptococcus neoformans. Med Mycol 34:385–391. doi:10.1080/02681219680000691 8971627

[19] McDade HC, Cox GM. 2001. A new dominant selectable marker for use in Cryptococcus neoformans*.* Med Mycol 39:151–154. doi:10.1080/mmy.39.1.151.154 11270405

[20] Wang P. 2021. Genetic transformation in Cryptococcus species. J Fungi 7:56. doi:10.3390/jof7010056 PMC7829943 33467426

[21] García-Nafría J, Watson JF, Greger IH. 2016. IVA cloning: a single-tube universal cloning system exploiting bacterial in vivo assembly. Sci Rep 6:27459. doi:10.1038/srep27459 27264908 PMC4893743

[22] Lin J, Fan Y, Lin X. 2020. Transformation of Cryptococcus neoformans by electroporation using a transient CRISPR-Cas9 expression (TRACE) system. Fungal Genet Biol 138:103364. doi:10.1016/j.fgb.2020.103364 32142753 PMC7153975

[23] Becker DM, Guarente L. 1991. [12] High-efficiency transformation of yeast by electroporation, p 182–187. In Methods in enzymology. Elsevier. 10.1016/0076-6879(91)94015-5 2005786

[24] Fan Y, Lin X. 2018. Multiple applications of a transient CRISPR-Cas9 coupled with electroporation (TRACE) system in the Cryptococcus neoformans species complex. Genetics 208:1357–1372. doi:10.1534/genetics.117.300656 29444806 PMC5887135

[25] Fernandes KE, Fraser JA, Carter DA. 2022. Lineages derived from Cryptococcus neoformans type strain H99 support a link between the capacity to be pleomorphic and virulence. MBio 13:e0028322. doi:10.1128/mbio.00283-22 35258331 PMC9040854

[26] Brilhante RSN, España JDA, de Alencar LP, Pereira VS, Castelo-Branco D de SCM, Pereira-Neto W de A, Cordeiro R de A, Sidrim JJC, Rocha MFG. 2017. An alternative method for the analysis of melanin production in Cryptococcus neoformans sensu lato and Cryptococcus gattii sensu lato. Mycoses 60:697–702. doi:10.1111/myc.12650 28699287

[27] García-Rodas R, Trevijano-Contador N, Román E, Janbon G, Moyrand F, Pla J, Casadevall A, Zaragoza O. 2015. Role of Cln1 during melanization of Cryptococcus neoformans. Front Microbiol 6:798. doi:10.3389/fmicb.2015.00798 26322026 PMC4532930

[28] Costas AMG, White AK, Metcalf WW. 2001. Purification and characterization of a novel phosphorus-oxidizing enzyme from Pseudomonas stutzeri WM88. J Biol Chem 276:17429–17436. doi:10.1074/jbc.M011764200 11278981

[29] Upadhya R, Lam WC, Maybruck BT, Donlin MJ, Chang AL, Kayode S, Ormerod KL, Fraser JA, Doering TL, Lodge JK. 2017. A fluorogenic C. neoformans reporter strain with a robust expression of m-cherry expressed from a safe haven site in the genome. Fungal Genet Biol 108:13–25. doi:10.1016/j.fgb.2017.08.008 28870457 PMC5681388

[30] Arras SDM, Chitty JL, Blake KL, Schulz BL, Fraser JA. 2015. A genomic safe haven for mutant complementation in Cryptococcus neoformans. PLoS One 10:e0122916. doi:10.1371/journal.pone.0122916 25856300 PMC4391909

[31] Figueroa IA, Coates JD. 2017. Microbial phosphite oxidation and its potential role in the global phosphorus and carbon cycles, p 93–117. In Advances in applied microbiology. Elsevier. 10.1016/bs.aambs.2016.09.004 28189156

[32] Achary VMM, Ram B, Manna M, Datta D, Bhatt A, Reddy MK, Agrawal PK. 2017. Phosphite: a novel P fertilizer for weed management and pathogen control. Plant Biotechnol J 15:1493–1508. doi:10.1111/pbi.12803 28776914 PMC5698055

