# Supplementary material for: Development of ptxD/Phi as a new dominant selection system for genetic manipulation in Cryptococcus neoformans
Source: Microbiol Spectr. 2024 Nov 20;13(1):e01618-24. doi: 10.1128/spectrum.01618-24 (PMC11705812; doi:10.1128/spectrum.01618-24)
Supplement: Supplemental material — Tables S1 to S4; Fig. S1 to S4. [file spectrum.01618-24-s0001.pdf]

SUPPLEMENTARY INFORMATION

Development of *ptxD*/Phi as a new dominant selection system for genetic manipulation in *Cryptococcus neoformans*

Muthita Khongthongdam, Tanaporn Phetruen, Sittinan Chanarat*

Laboratory of Molecular Cell Biology, Department of Biochemistry and Center for Excellence in Protein and Enzyme Technology, Faculty of Science, Mahidol University, Bangkok, Thailand

Corresponding author: sittinan.cha@mahidol.edu

Table S1. The yeast strain was used in this study.

Strain	Genotype	Reference
C. neoformans KN99	*MATα*	BEI Resources
C. neoformans KN99α	*MATα* SH2::*ptxD*	This study
C. neoformans KN99α	*MATα Δade2::ptxD*	This study

Table S2 Plasmid used in this study.

Plasmid name	Description	Reference
pCnCas9:U6-gRNA	A *U6* promoter driving the expression of the single guide RNA (sgRNA) or fusion PCR.	Wang (2018)
pBHM2329	A scaffold and *6T* terminator for fusion PCR.	Huang et al. (2021)
pBHM2403	pRS316-*pTEF1*-CnoCas9, *C. neoformans* codon-optimized Cas9.	Huang et al. (2021)
pFA6a *hphNT1*	Contains *hphNT1* gene, providing resistance to Hygromycin B for selection of plasmid-incorporated cells.	http://www.euroscarf.de/plasmid_details .php?accno=P30347
pESC-URA	Company-synthesized plasmid containing codon-optimized *ptxD* gene for *C. neoformans*.	This study
pFA6a Cno *ptxD*	Derived from cloning to enable expression of *ptxD* gene in *C. neoformans.*	This study

Table S3 Primers used in this study.

Primers		Sequences (5'-3')
Vector_pFA6a_*hphNT1*_F		F' = GAGGGCAAAGGAATAATCTCG
Vector_pFA6a_*hphNT1*_R		R' = GGTTGTTTATGTTCGGATGTGA
Insert_*ptxD*_F		F' = ACATCCGAACATAAACAACCGAATTCATGCTT CCAAAACTTGT
Insert_*ptxD*_R		R' = GAGATTATTCCTTTGCCCTCTCAGATCTTATC GTCGTCATCC
*CAS9*_F		F' = ATTGTAATACGACTCACTATAGGG
*CAS9*_R		R' = TCGAGGTCGACGGTATCGAT
sgRNA (*SH2*)	*SH2*_A_F	F' = TGCATTAGAACTAAAAACAAAGC
	*SH2*_A_R	R' = AGACTCCACAGCCTAAGATCAACAGTATAC CCTGCCGGT
	*SH2*_B_F	F' = GATCTTAGGCTGTGGAGTCTGTTTTAGAGC TAGAAATAGCAAGT
	*SH2*_B_R	R' = CGTCCTGTCACATCTTACC
sgRNA (*ADE2*)	*ADE2*_A_F	F' = TGCATTAGAACTAAAAACAAAGC
	*ADE2*_A_R	R' = TGTTCCTAACGACCATAACCAACAGTATACC CTGCCGGT
	*ADE2*_B_F	F' = GGTTATGGTCGTTAGGAACAGTTTTAGAGCT AGAAATAGCAAGTT
		R' = CGTCCTGTCACATCTTACC
*ptxD*_F		F' = CGTACGCTGCAGGTCGAC
*ptxD*_R		R' = ATCGATGAATTCGAGCTCG
Colo_*ptxD*_F		F' = GCGGATGGCGTACAGC
Colo_*ptxD*_R		R' = ATTTCAGACAATTGTAGAGACCG

Table S4. Transformation efficiency of *ptxD* to SH2 and *ADE2* loci.

Strain	*ptxD* construct (μg)	Cas9 (μg)	sgRNA (μg)	Number of colonies (Mean ± SD)
SH2::*ptxD*	2	0.7	0.7	24 ± 12.12
Δ*ade2::ptxD*	2	0.7	0.7	7.33 ± 4.04

Supplementary Figure 1

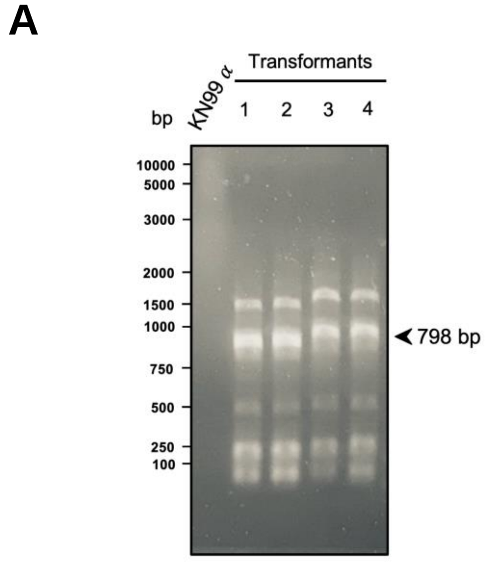

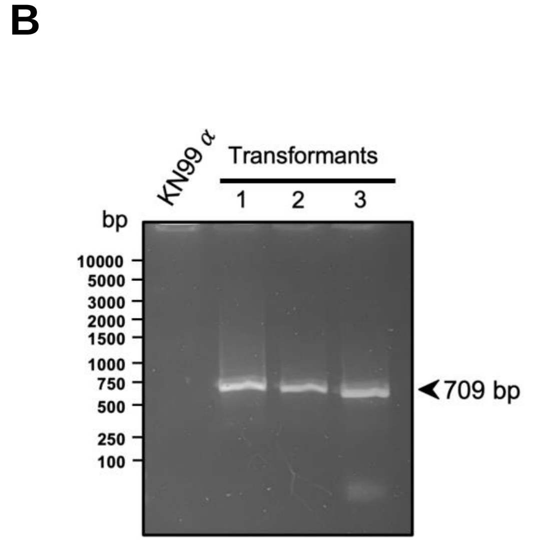

Figure S1. Colony PCR analysis confirming the successful integration of the *ptxD* gene. (A) Similar to Figure 3C, but with different primers: the forward primer annealing outside the *ptxD* gene insert at the SH2 locus, and the reverse primer annealing within the integration construct. **(B)** Similar to S1A, but with the forward primer annealing at the *ADE2* locus.

Supplementary Figure 2

A

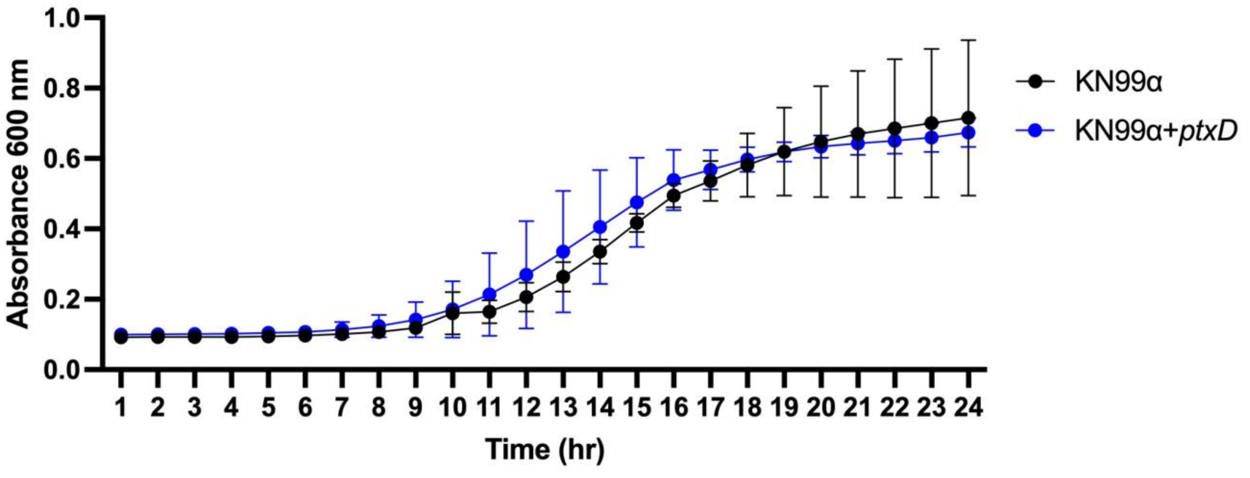

B

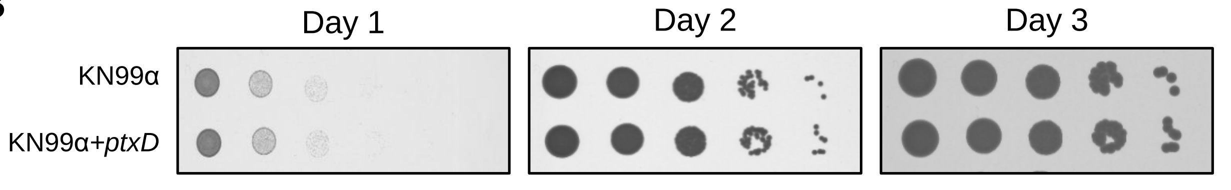

Figure S2. Effect of *ptxD* integration on *C. neoformans* in SC all. (A) Growth curves of *C. neoformans* strain KN99α in SC all media. Cultures of the *ptxD* integrated strain and the control strain KN99α were grown in YPD medium, and optical density (OD_{600}) was measured every 1 hour over 24 hours. **(B)** Growth of *C. neoformans* strains on SC all agar plates. The *ptxD* integrated strain and control strain KN99α colonies were spotted on SC all agar plates and incubated at 30°C 2for 3 days. There was no significant difference in growth between the *ptxD* integrated strain and the control strain KN99α in the SC all medium.

Supplementary Figure 3

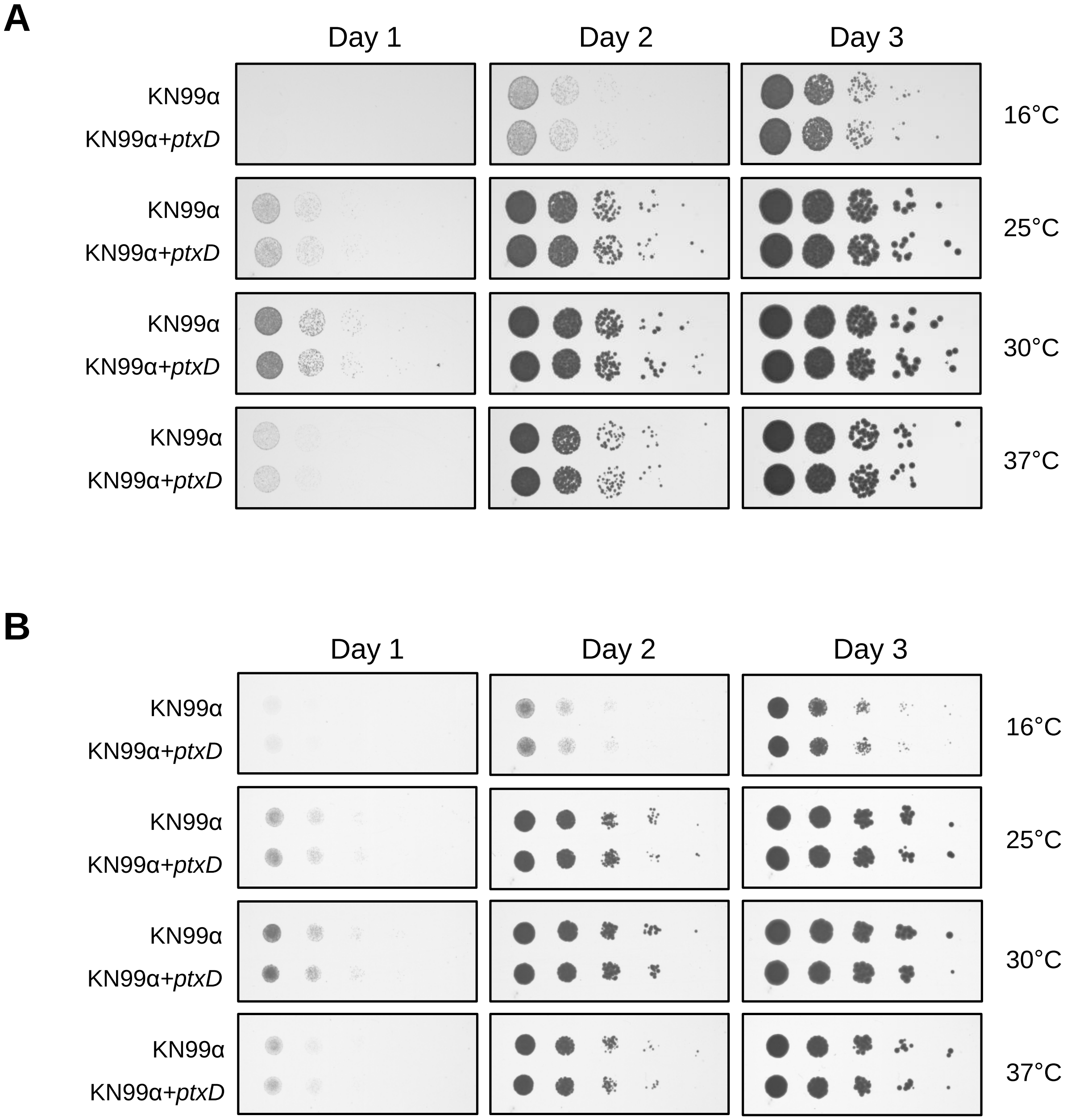

Figure S3. Effect of temperature on the growth phenotype of wild-type KN99α and the *ptxD*-expressing strain. Dot spot analysis of wild-type KN99α and a clone with *ptxD* gene integrated at the SH2 locus on YPD **(A)** and SC all **(B)** media. Plates were incubated at 16°C, 25°C, 30°C and 37°C for 3 days.

Supplementary Figure 4

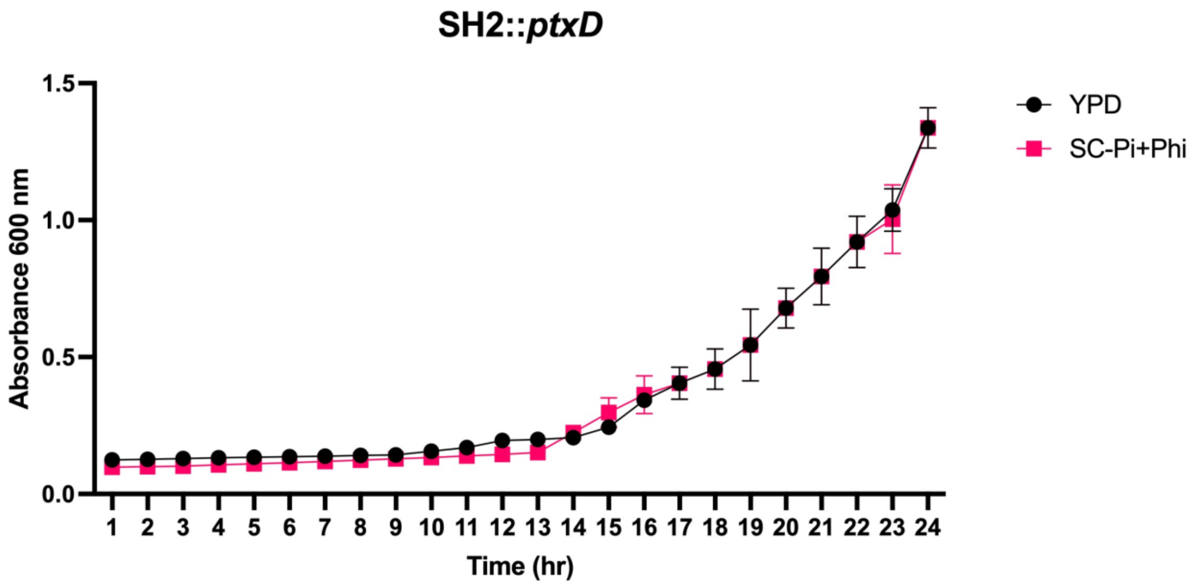

Figure S4. Growth curve analysis showing the doubling times of *C. neoformans* strain KN99α expressing *ptxD* gene in SC-Pi+Phi media compared to YPD.
